# Conservation of Giant Honey Bee (*Apis dorsata* F.) for Honey and Beeswax Production and Sustainable Pollination Services

**DOI:** 10.3390/insects16060560

**Published:** 2025-05-26

**Authors:** Ram Chander Sihag

**Affiliations:** Laboratory of Animal Behavior & Simulation Ecology, Department of Zoology, College of Basic Sciences & Humanities, CCS Haryana Agricultural University, Hisar 125004, India; rcsihag@hau.ac.in or rcsihag@gmail.com

**Keywords:** *Apis dorsata*, conservation, domestication, giant honey bee, pollination services, sustainability

## Abstract

During the past quarter-century, the natural populations of giant honey bees (*Apis dorsata*) have declined markedly. The loss of nesting sources is one of the many reasons for its decline. This has threatened the pollination services of several agricultural and wild plants in areas of its natural habitat. To sustain pollination services in the natural habitat of this honey bee, conservation efforts are crucial. For this purpose, 1 m-long, 15 cm-wide, and 0.5 m-deep wooden nesting planks were designed and prepared. The latter were hung on the eaves/projections of university buildings to attract the migratory swarms of this honey bee. The occupancy of these planks confirmed that the bees accepted them as nesting sites. The experimental trial was conducted with four treatments, each replicated four times over a period of 10 years. This honey bee accepted, occupied, and nested on only those wooden planks that were treated with molten beeswax or contained traces of beeswax and were tightly fixed to the surface of the eaves/building projections. The occupation and re-occupation indices revealed that this honey bee had strong site preferences. Spraying water on live colonies was the most effective method for safely handling this honey bee, while smoking was slightly less effective. Frequently disturbed colonies showed less aggressiveness than the undisturbed colonies. This study aids in the domestication, handling, and conservation of this honey bee in its natural habitat, where the continuous removal of nesting sources threatens the sustainability of pollination services.

## Simple Summary

The giant honey bee (*Apis dorsata*) is a superb pollinator of agricultural crops and a magnificent producer of honey and beeswax. Its population has drastically decreased in recent years. This has impacted the production of honey and beeswax, as well as threatened local pollination services. This study explores methods to domesticate and safely handle it to maximize its potential for these services. These innovative approaches would help preserve this honey bee to produce honey and beeswax and ensure sustainable pollination services in areas of its natural habitat.

## 1. Introduction

The giant honey bee (*Apis dorsata* F.) is an economically important crop pollinator in Pakistan, South Asia, and Southeast Asia [1,2,3,4,5]. This honey bee inhabits the Asian tropics, where its colonies produce substantial honey and beeswax in a single, large comb [1,4]. In recent years, the number of colonies of this honey bee has markedly declined [2]. The main reason for such a decline is the loss of nesting sources and traditional destructive methods of honey hunting. The latter practice involves smoking and burning colonies, which leads to comb destruction and large-scale bee mortality. It has been suggested that obtaining good-quality honey from *A. dorsata* using traditional methods of honey harvesting was not a desirable technique [6]. This honey-hunting practice leads to the mass destruction of colonies, thereby negatively impacting local pollination services. For the sustainability of pollination services and the safe honey harvest from this honey bee, proper domestication, handling, and conservation of this honey bee have become increasingly important. This necessity prompted the undertaking of these investigations.

This honey bee exhibits migratory behavior. Many colonies of this honey bee migrate seasonally, living in two or more rich forage areas in the course of each year [7,8,9,10,11,12]. In the semi-arid environments of Northwest India, the migratory swarms of this honey bee arrive in October/November, remain there to reproduce and form swarms, and then emigrate in mid-May [2]. In the southernmost provinces in Vietnam, Minh Hai, Kien Giang and Hau Giang, which are west of the Mekong River, *A. dorsata* migrates between mangrove forests on the coast and the swamp forests of *Melaleuca leucadendron* farther inland, which seasonally produce much pollen and nectar [1].

Recent reports have revealed that these honey bee colonies in the semi-arid environments of Northwest India make nests on the cliffs/projections of multi-story buildings and the branches of tall trees. This honey bee has some fixed preferences for many nesting alternatives, including a preference for the nesting place/site, the height of the nesting site, and the direction of the nest. These parameters seemed to play a decisive role in the selection of the source and the site for nesting [13]. For example, *A. dorsata* prefers smooth surfaces over uneven ones for nesting. The majority of colonies nest at heights between 14 and 17 m on supports with an inclination from 0° to 45°, orient their nests in an east–west direction and choose a site with relics of an abandoned nest. Most newly arrived swarm colonies construct nests measuring 100–120 cm in length and 30–50 cm in height. The basal thickness of the comb in the non-honey region is 2.04 ± 0.6 cm, while in the honey region, it is 5.7 ± 1.2 cm [13].

This honey bee has long faced predation from humans, who employ destructive honey-hunting methods. Therefore, there is a need to develop a non-destructive method of honey hunting for this species. Not many reports are, however, available on the domestication and conservation of this honey bee. Rafter beekeeping with *A. dorsata* is known in some southeastern countries [14,15,16,17,18,19]. However, this practice was conducted in areas where natural nesting sources for this honey bee are abundant. No such efforts have been made in areas where nesting sources of this honey bee have been depleted due to man-engineered activities. Some efforts have also been made towards the hiving of this honey bee [6,20] but with limited success. Some ‘attraction planks’ to capture migrating *A. dorsata* swarms were also developed and tested in Southwest India [6]. The researcher claimed that his device improved honey harvesting from this species; however, the method was never widely adopted.

The foregoing information clearly reveals that this honey bee is a key provider of honey, beeswax, and pollination services in areas of its natural habitat. Ensuring the continued availability of these services requires the domestication and conservation of this species. This study was conducted with the following four objectives: (i) examining the role of the giant honey bee (*A. dorsata*) as a crop pollinator, (ii) testing nesting devices for attracting and domesticating its migratory swarms, (iii) developing its safe handling methods, and (iv) evaluating its potential for honey and beeswax production.

## 2. Material and Methods

**Figure 1:**
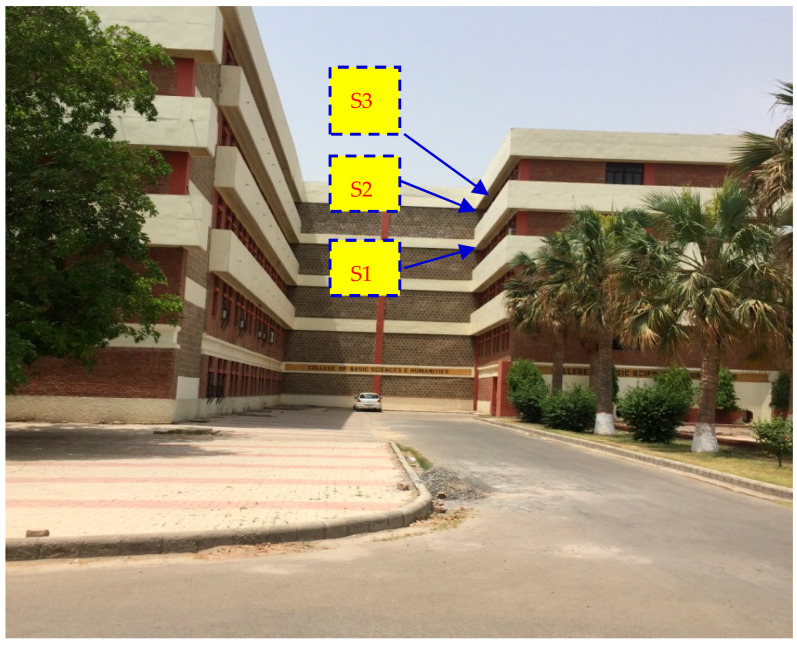
The multi-story building of the College of Basic Sciences and Humanities, where *A. dorsata* nested on various ceiling projections (Adapted from Sihag, 2017 [13]). S1, S2 and S3 represent the Site-1, Site-2 and Site-3 where nesting planks were hung. Site -4 and Site -5 were on the projection of rear left wing (see Figures 8 and 12), and Site-6 was on the projection of rear right wing of the building (see Figure 3).

This study took place at the College of Basic Sciences and Humanities, Haryana Agricultural University, Hisar, India (Figure 1), where different types of observations on *A. dorsata* were documented and listed in the following subchapters.

### 2.1. Apis dorsata as a Pollinator of Crops

Year-round surveys have been conducted over the past 40 years (1984–2024) to identify the crop plants visited by *A. dorsata* foragers in Hisar, India. Foraging behavior was recorded using established methods [21,22]. It was assessed whether the foragers functioned as pollinators or nectar thieves on the flowers they visited. Based on these observations, their contribution to regional pollination services was evaluated.

### 2.2. Trials for Domestication and Conservation of A. dorsata

**Figure 2:**
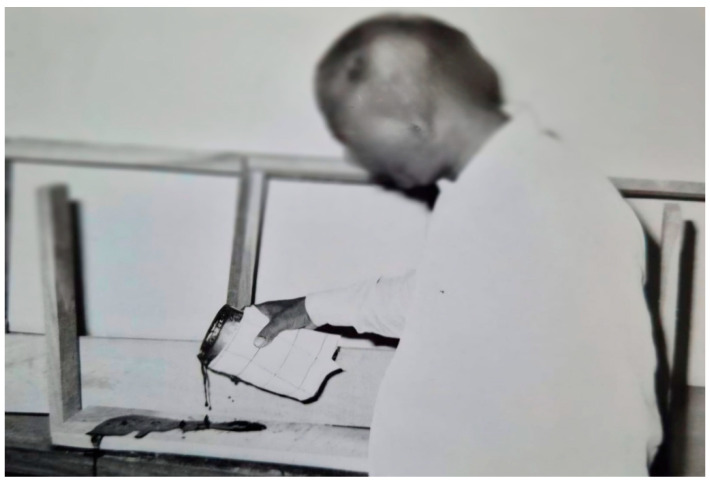
Wooden planks used for the domestication of *A. dorsata*; molten beeswax is applied to the underside of the plank.

The primary nesting sources of A. dorsata in the study region were identified. Drawing on previous research [13], nesting planks made from Acacia nilotica wood were designed and built, measuring 1 m in length, 15 cm in width, and 0.5 m in depth (Figure 2). Molten beeswax was applied to the underside of the treated planks, while control planks were left uncoated (Figure 2).

**Figure 3:**
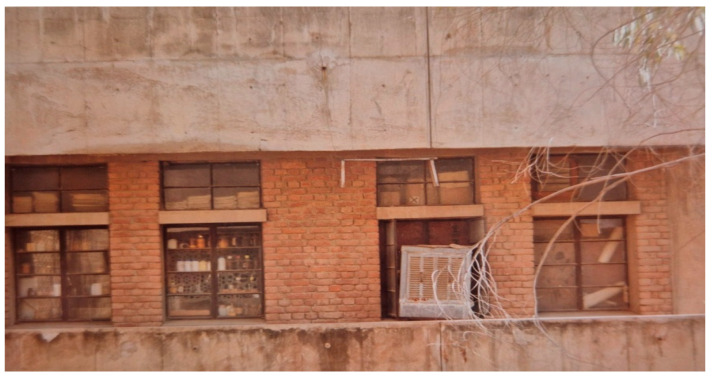
A loosely tied wooden plank hung on the projection of the building using thin iron wires (Site-6).

These planks were hung before the arrival of swarms from the eaves of the building of the College of Basic Sciences and Humanities, Haryana Agricultural University (Hisar, India), using thin iron wires (Figure 3). These planks were subjected to the following four treatments before testing:

(i) Planks treated with beeswax loosely tied to the building eaves.
(ii) Planks treated with beeswax tightly tied to the building eaves.
(iii) Untreated planks loosely tied to the building eaves.
(iv) Untreated planks tightly tied to the building eaves.

The planks were hung in the preferred direction and height before the arrival of migratory swarms of *A. dorsata* in this region [13]. Each treatment was replicated four times (four planks were used for each treatment), and the experiment ran for 10 years (1984 to 1993). Therefore, each treatment had 40 replications during those 10 years. The acceptance of the wooden planks was confirmed on the basis of their occupancy by the migratory swarm colonies of the giant honey bee (*A. dorsata*).

### 2.3. Occupation and Re-Occupation Indices

Six nesting sites were selected to test the preferred locations for migratory swarms of *A. dorsata*. On each site, one beeswax-treated plank was hung and tightly tied to the building’s projection in 1994, and the experiment continued until 2012 (for 19 years).

The occupation and re-occupation indices were determined by the following equations:

(1) $$OI=\frac{n}{N}$$

where

OI = Occupation index.

n = Number of years when the plank was available for the first occupation.

N = Number of years when the plank was actually occupied.

(2) $$ROI=\frac{m}{M}$$

where

ROI = Re-occupation index.

m = Number of times when the plank was utilized for re-occupation.

M = Number of times when the plank was actually available for re-occupation.

### 2.4. Trials for Handling and Taming of A. dorsata Colonies

These trials were performed on a bright day using colonies of this honey bee that had already settled on the artificial nesting planks. Two kinds of trials were performed, and the aggressive response of the colonies was tested in the following two ways:

#### 2.4.1. Pre-Handling Disturbance Trials

This experiment started on colonies after a month of their settlement on the nesting planks. In this experiment, colonies were divided into two groups: (i) periodically disturbed colonies and (ii) undisturbed (control) colonies. Periodic disturbance to the colonies occurred at a 10-day interval (i.e., first on the 40th, second on the 50th, third on the 60th, fourth on the 70th, and fifth on the 80th day after settlement). The same five colonies were repeatedly subjected to the disturbance treatment. However, the control colonies did not receive any disturbance before handling, and every time fresh colonies were selected for this purpose. In the control colonies, an aggressive response was recorded after the 40th day in five colonies, after the 50th day in another five colonies, and so on. This trial could not be completed in the same year due to the limited number of colonies, and the experiment was extended over multiple years. The disturbance to the colonies was caused by loosening the wire of the planks carrying the colonies, waving them gently up and down and to and fro in all directions, giving them minor jerks, and going near these colonies. The final aggressive response of the two types of colonies was recorded when approaching the colony and physically touching the curtain of bees on the comb. The intensity of aggressiveness of the two types of experimental colonies was recorded, as shown in Table 1.

#### 2.4.2. Pre-Handling Taming Trials

In these trials, the colonies were divided into four groups: (i) smoke-treated colonies, (ii) water-treated colonies, (iii) sham-treated colonies, and (iv) untreated (control) colonies. For the smoke treatment, 15–20 gentle puffs of smoke were applied to the experimental colonies using a smoker. Jute cloth was burned in the smoker to produce smoke. In the water-treated colonies, normal clean water at room temperature was sprayed on the two faces of the colony using a one-liter hand sprayer with a fine nozzle. The sham treatment was given by gently making 15–20 air puffs on the colonies. The control colonies did not receive any taming treatment. Two types of colonies were selected for this experiment: the undisturbed colonies and previously disturbed colonies. For these trials, the colonies were lowered to chest height, and precautions were taken to minimize disturbances aside from the experimental treatments. The aggressive responses of the colonies subjected to the four treatments were recorded (see Table 1).

### 2.5. Utilization of the Attracted A. dorsata for Honey and Beeswax Production and for Live Studies

**Figure 4:**
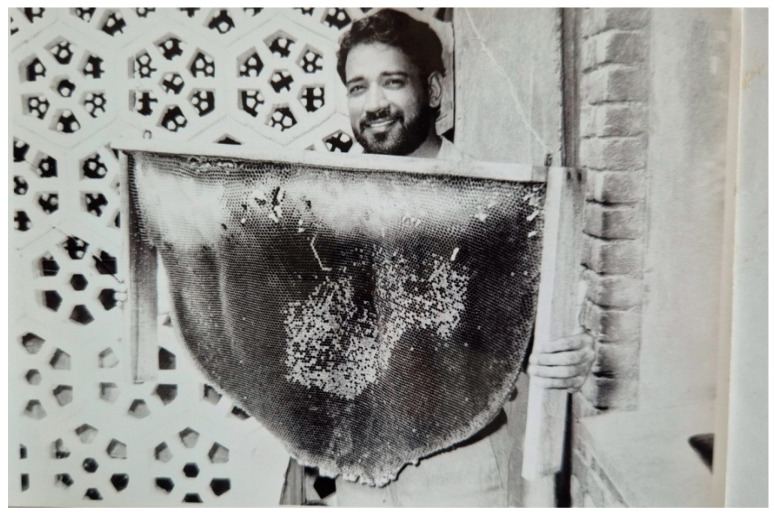
An abandoned comb of *A. dorsata*, which was raised on the wooden plank; the white upper portion shows the honeycomb; sealed brood is located in the center, and queen cells are located at the lower end (comb frame held by the author). Beeswax was recovered from an empty comb using conventional methods.

**Figure 5:**
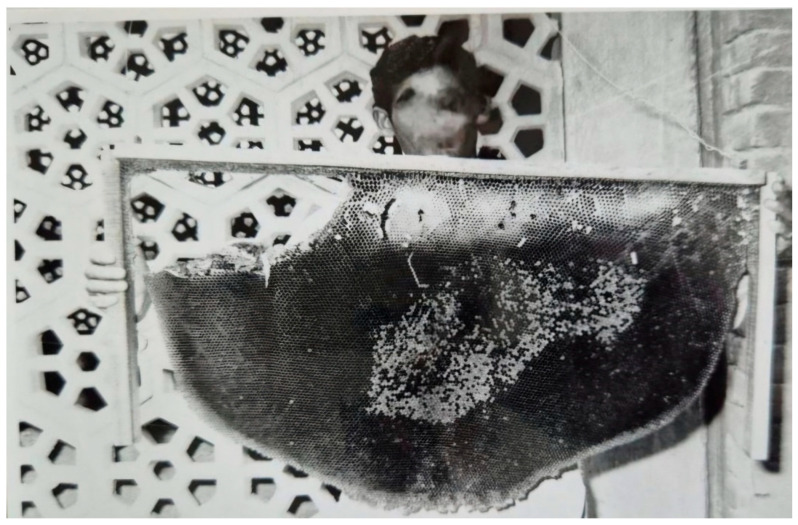
A comb of *A. dorsata*; the honey portion was excised from the upper left portion. The deserted comb makes an excellent source of beeswax and can be used for research.

A method was developed to use colonies of *A. dorsata* that had been attracted and tamed for honey and beeswax production, as well as for live studies. For this purpose, the colonies were periodically disturbed, as described in Section 2.4.1. Afterward, the colonies were brought down and suitably tamed following the procedures outlined in Section 2.4.2. The honey portion of the comb was then manually excised using a knife. The excised honeycomb was subsequently squeezed by hand to extract the honey. Beeswax was then recovered from the abandoned combs using conventional methods (Figure 4 and Figure 5). In total, honey and beeswax production from 10 domesticated colonies was recorded. The potential of this species to produce honey and beeswax was assessed. The attracted colonies were also used for live studies of this honey bee [2,13].

### 2.6. Statistical Analysis

For the domestication trials, no statistical tests were required [23]. However, for the handling and taming trials, an unpaired *t*-test [24] and one-way ANOVA [25] were used, as appropriate, to find differences between trials.

## 3. Results

### 3.1. Apis dorsata as an Important Pollinator of Crops

**Figure 6:**
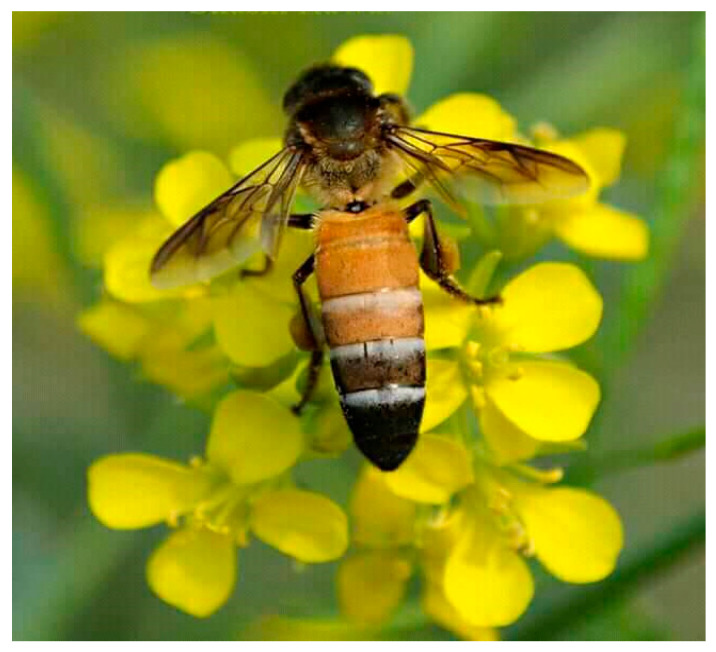
*Apis dorsata* foraging on rapeseed (*Brassica campestris*) flowers for pollen and nectar; the forager always served as a pollinator by transferring pollen during each foraging visit [21,22].

**Figure 7:**
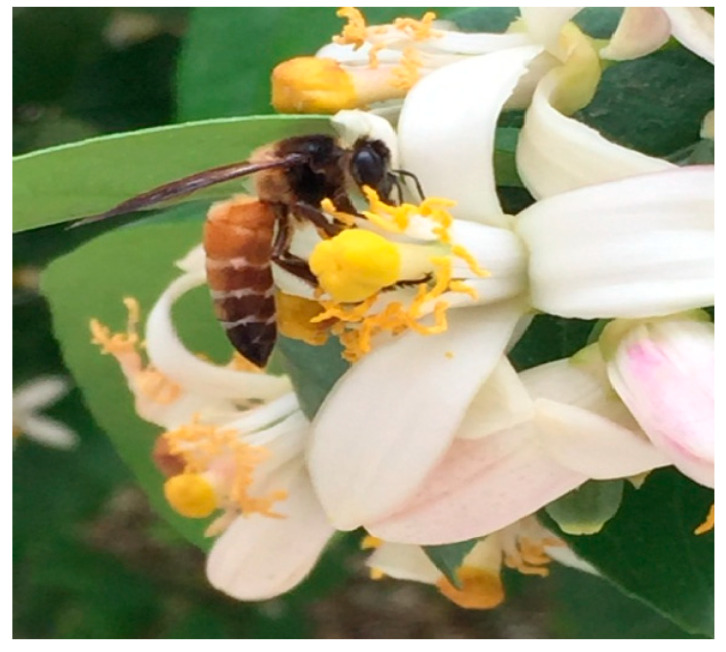
*Apis dorsata* foraging on lemon (*Citrus limon*) flowers for pollen and nectar; the forager always served as a pollinator by transferring pollen during each foraging visit [21,22].

In Hisar, the foragers of *A. dorsata* visited the flowers of more than 30 crops for pollen and nectar (Figure 6 and Figure 7; Table 2). These foragers transferred pollen to each visited flower, thus facilitating pollination. Based on the foraging behavior, this honey bee is documented as an important pollinator of the crops grown in this region.

### 3.2. The Nesting Sources of A. dorsata

**Figure 8:**
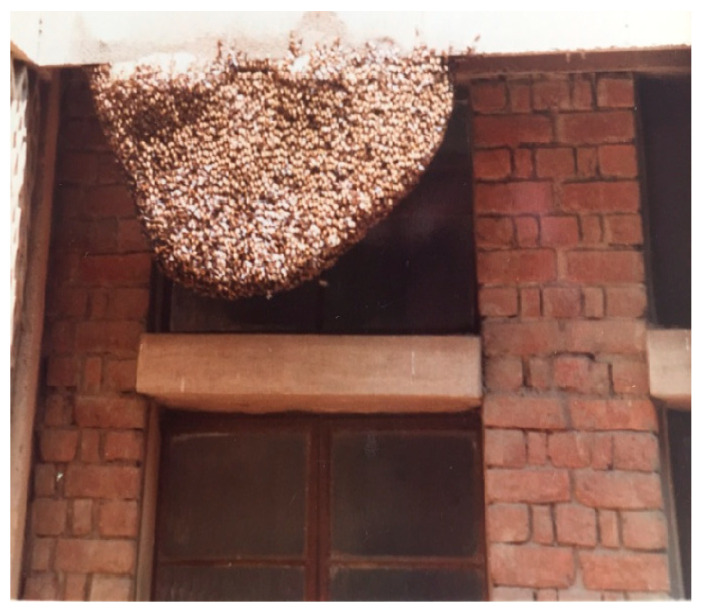
A colony of *A. dorsata* nesting on the college building’s projection, which is roughly 15 m above the ground (Adapted from Sihag, 2017 [13]) (Site-4).

**Figure 9:**
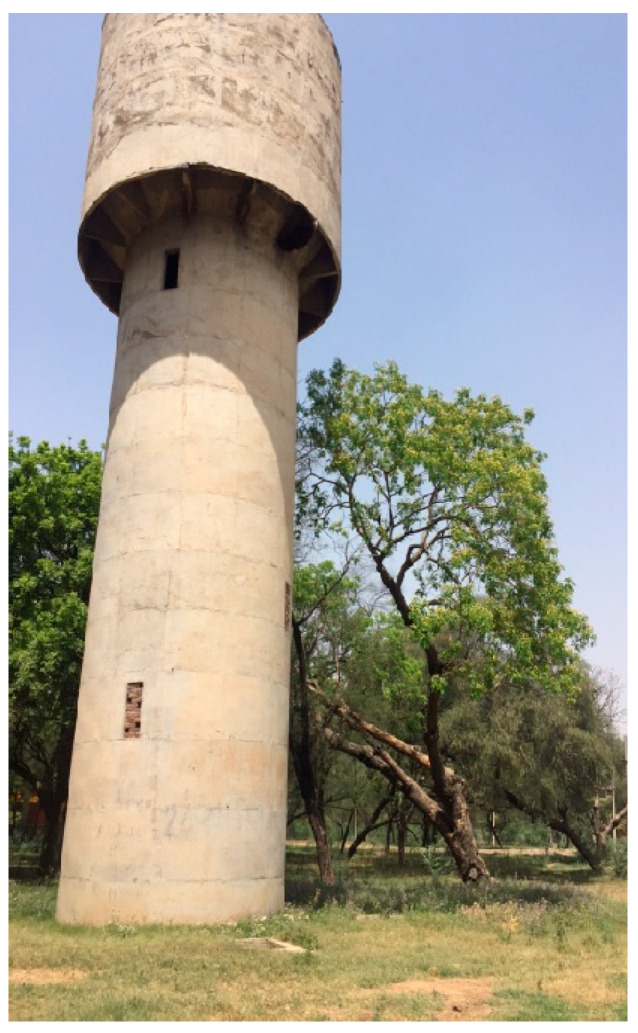
Colonies of *A. dorsata* nesting on the water tower building, which is roughly 15 m above the ground (Adapted from Sihag, 2017 [13]).

**Figure 10:**
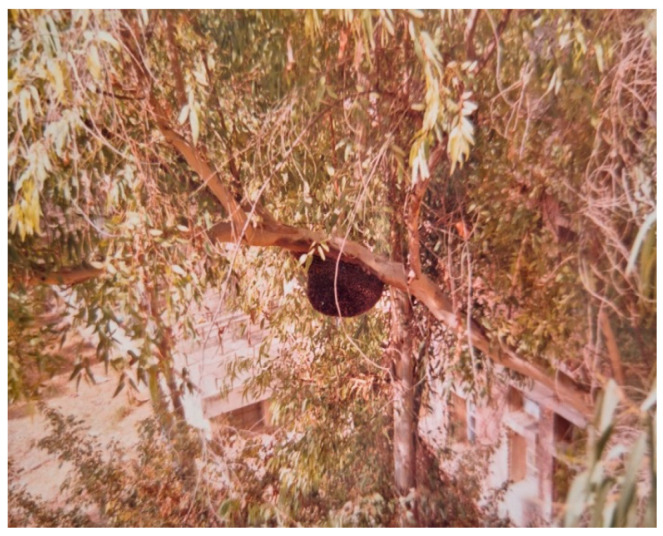
A colony of *A. dorsata* nesting on a eucalyptus tree roughly 5 m above the ground.

In Hisar, *A. dorsata* has very few places to build nests. These include man-made structures like tall buildings and water towers (Figure 8 and Figure 9) as well as tall trees like Indian rosewood (*Dalbergia sissoo*), eucalyptus (*Eucalyptus hybrida*), Indian banyan (*Ficus benghalensis*), and religious peepal (*Ficus religiosa*) (Figure 10).

**Figure 11:**
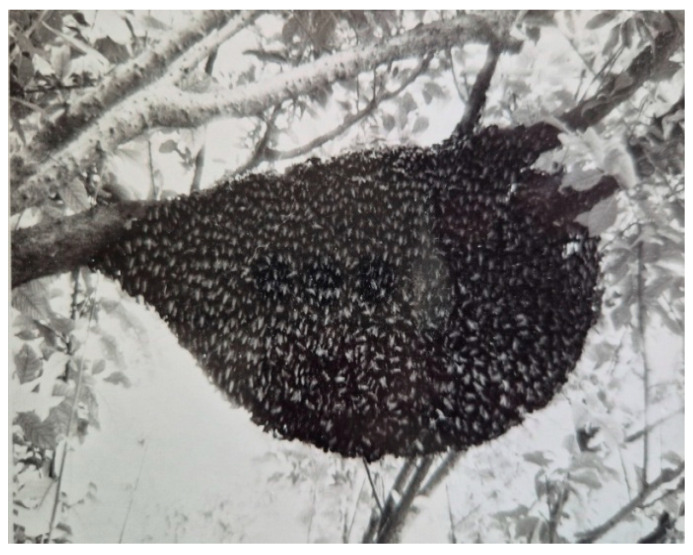
A colony of *A. dorsata* nesting on a jujube tree roughly 5 m above the ground.

Occasionally, dwarf trees planted in undisturbed areas, such as horticultural fields, also serve as nesting sources for this honey bee (Figure 11). Buildings continue to be the best alternative sources for hanging the nesting planks to attract the migratory swarms of this honey bee because host nesting sources are being quickly removed from this area [2].

### 3.3. Trials for Domestication and Conservation of A. dorsata

**Figure 12:**
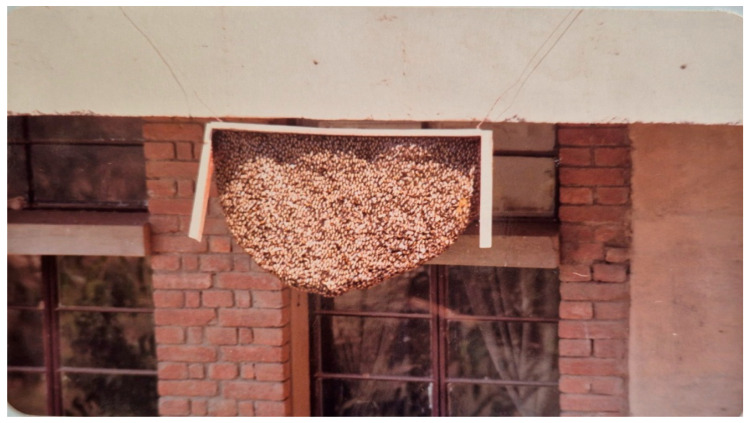
A colony of *A. dorsata* that built its nest on a beeswax-treated wooden plank. The colony has been brought down to conduct various experiments by loosening the plank’s wire (Site-5).

**Figure 13:**
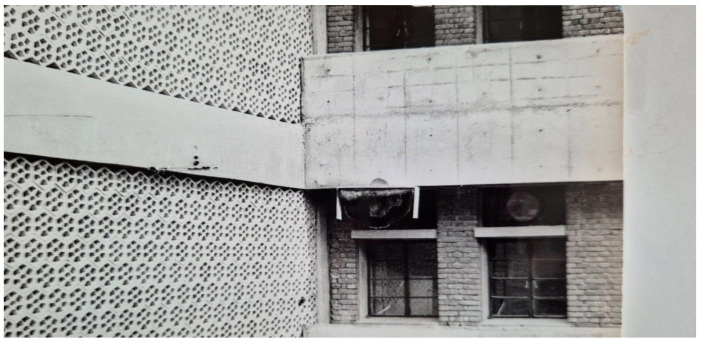
An abandoned *A. dorsata* comb constructed on a wooden plank that was tightly tied to the building projection (Site-1).

For the artificial devices, this honey bee had a strong choice. Of the four treatments tested, three were completely ignored or rejected (Table 3). These included (i) beeswax-treated planks tied loosely to the building eaves, (ii) untreated planks tied loosely to the building eaves, and (iii) untreated planks tied tightly to the building eaves. Only the beeswax-treated planks tied tightly to the building eaves were accepted (Figure 12 and Figure 13). However, only 29 of the 40 trials were occupied, indicating that the occupancy rate of the provided planks was moderate (58%). After remaining there from October to May, the nested colonies eventually left.

### 3.4. Occupation and Re-Occupation Indices

**Figure 14:**
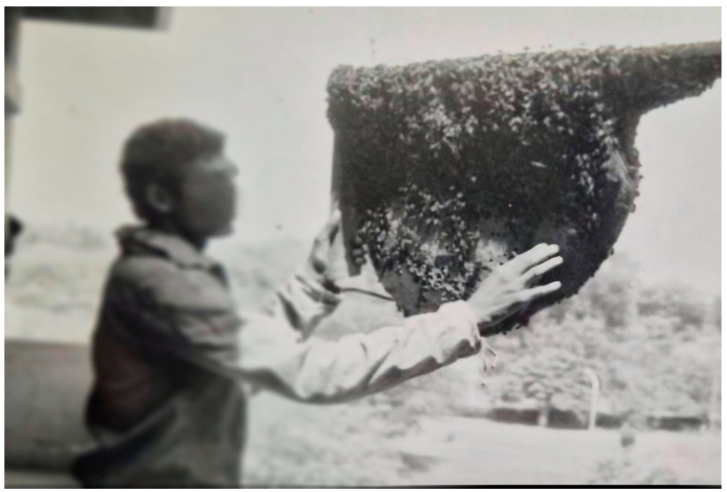
A live colony of *A. dorsata*. The honey portion was excised following the taming trials, and the colony was readily manageable for live research (Site-2).

Interestingly, both the occupation and re-occupation indexes for Site 1 (Figure 1) and Site 4 (Figure 13) were equal to one. These honey bees completely occupied and reoccupied the planks that were hung on these sites year after year (Table 4). Site 2′s (Figure 14) occupation index was equal to one, meaning it was used in the first year it became available; however, its re-occupation index was 0.5 and was only used 9 of the 18 times it was available. The planks hung at Site 3 had an occupation index equal to 0.5; they were used in the second year of their availability. However, their re-occupation index was 0.29, i.e., planks were only used 5 out of the 17 times they were available.

Similarly, Site 5′s (Figure 8) planks had an occupation index of 0.33 (occupied in the third year of availability) but a significantly higher re-occupation index of 0.5 (used eight times out of 16 times of availability), suggesting that the honey bees moderately preferred this site. With a 0.2 occupation index (used in the fifth year after becoming available) and a re-occupation index (used only 3 times despite being available 14 times), the nesting plank hung at Site 6 (Figure 6) demonstrated very little attractiveness to the giant honey bee swarms (Table 4). These findings suggest that these honey bees did not find all of the nesting locations to be equally attractive and that they preferred some over others based on the availability of nesting locations.

### 3.5. Trials for Handling and Taming of A. dorsata Colonies

#### 3.5.1. Pre-Handling Disturbance Trials

The aggressive response of the test colonies under the two types of trials is displayed in Table 5. Colonies that had not been disturbed before exhibited a significantly higher level of aggression than those that had been disturbed frequently (*p* < 0.0001, *t*-test value = 7.56; df = 48, Table 5).

The often disturbed colonies began ignoring the surrounding disturbances and progressively became quiet and calm in the presence of visitors. In contrast, the undisturbed colonies exhibited a very high degree of aggressiveness even after 80 days of their settlement if they were not frequently disturbed, as they continued to exhibit exceptionally high aggression (Table 5). The difference in aggressiveness between days was highly significant (F_4, 24_ = ∞, ANOVA, Table 5).

#### 3.5.2. Pre-Handling Taming Trials

The colonies of *A. dorsata* exposed to four different types of taming treatments responded differently (Table 6).

Both previously disturbed and undisturbed colonies’ levels of aggression were affected by these taming trials in the same way (*p* > 0.05, *t*-test value = 0.238, df = 38, Table 6). However, in both previously disturbed and undisturbed colonies, the difference between taming trials was highly significant (F_3, 19_ = ∞, ANOVA, Table 6). Undisturbed colonies treated with smoke were slightly more aggressive than those treated with water. Colonies treated with smoke and water that had previously been disturbed, however, did not exhibit any different level of aggression. In actuality, these colonies were free of aggression. Sham-treated and control colonies—regardless of prior disturbance—displayed high levels of aggression. However, aggression was lower in previously disturbed colonies than in undisturbed ones. This lends further credence to earlier findings that *A. dorsata* aggression is reduced when the colonies are periodically disturbed. After being sprayed with water, neither disturbed nor undisturbed colonies displayed any sign of aggression (Table 6).

### 3.6. Utilization of the Attracted A. dorsata for Honey and Beeswax Production and for Live Studies

These honey bees produced 6.58 ± 0.53 kg (mean ± s.d.; N = 10) of honey and 1.52 ± 0.18 kg (mean ± s.d.; N = 10) of beeswax per colony on average (Table 7). The colonies attracted to the wooden planks also proved a convenient resource for conducting live research on the colony-life of this honey bee that would otherwise be extremely challenging to reach [2,14] (Figure 14).

## 4. Discussion

*Apis dorsata* is a vital part of the local pollination service because it has been documented as a dependable pollinator of over 30 crops cultivated in this area (Table 2). This honey bee produces a considerable amount of honey and beeswax (Table 7). No prior study has provided clear evidence for these characteristics of this honey bee. In recent times, the populations of this honey bee have decreased significantly due to the substantial loss of its nesting sources [2]. Thus, it is now absolutely necessary to conserve this honey bee. However, the conservation efforts must be coordinated with the migratory and nesting habits of this honey bee.

In India’s northwestern region, *A. dorsata* migrations are seasonal. Arriving in October or November, the honey bee colonies remain in this area throughout the autumn, winter, and spring before leaving in the summer [2]. These colonies construct their nests on the eaves of tall buildings and trees [13], as they do elsewhere [26,27,28]. In the past, many attempts were made to harvest the honey potential of this honey bee, mostly in the field of rafter beekeeping [14,15,16,17,18,19]. The majority of these attempts involved installing rafters on trees to serve as nesting sites for this honey bee. This strategy has become ineffective in areas where the natural nesting resources (tall trees) of this honey bee have been significantly removed [2]. The regeneration or restoration of these could take an extended period. Tall buildings can provide potential nesting sites during this period. Because of this, the nesting planks were hung on the building eaves at ideal heights and possible locations. For their acceptance, two criteria must be combined. These include: (i) the hanging plank’s underside is beeswax-coated, and (ii) the plank is perfectly aligned with the building projection’s surface. Despite their moderate acceptance, these efforts appeared to be rationally justified (Table 3). Furthermore, this study shows that this honey bee does not find all nesting sites equally appealing, and when given the option, it prefers some sites over others (Table 4). Therefore, before starting extensive beekeeping operations with this honey bee, initial efforts should be focused on identifying preferred sites for attracting migratory swarms.

Although this honey bee is used in rafter beekeeping, no efforts were made to attract and tame the colonies, and very little work was conducted to domesticate it [6,20]. This research provides methods for (i) attracting the migratory swarms of this honey bee to the nesting planks suspended from tall building projections and (ii) taming the colonies to lessen their aggressive behavior, getting close to them, and handling them gently for non-destructive honey harvesting and scientific research on the live colonies. Thus, the information this investigation provides is new to science. This research will open a new era of beekeeping with this honey bee for honey and beeswax production and the sustainability of pollination services in the areas of its natural habitat.

## 5. Conclusions

The giant honey bee (*A. dorsata*) has a vast potential for crop pollination and honey and beeswax production in the areas of its natural habitat. Because of a sharp decline in its nesting sources, its populations have drastically decreased. It is now critically important to conserve this honey bee. In order to attract and domesticate the swarms of this honey bee, artificial wooden planks were designed, built, and tested. Additionally, methods of safely handling the colonies were also investigated. This study will act as a guide for the conservation of this honey bee for honey and beeswax production and the sustainability of pollination services in areas of its natural habitats.

## Figures and Tables

**Table 1:** Level of aggression in *A. dorsata* caused by different taming trials.

Serial. Number	Degree of Aggression	Aggression Score
1	Spontaneous and very high aggression [Hundreds of bees attacked the person handling the colony]	5
2	Spontaneous and high aggression [Two to three hundred bees attacked the person handling the colony]	3
3	Delayed and mild aggression [Fewer than 100 (approximately 40–80) bees tried to attack the person handling the colony]	1
4	No aggression was observed [No bee attacks were observed]	0

**Table 2:** The various crops pollinated by *A. dorsata* in Hisar (India).

No.	Crop (Common Name)	Crop (Botanical Name)	Family	Flowering Months	Visit for
1	Onion	*Allium cepa* L.	Apiaceae	Mar.–Apr.	P, N
2	Sunflower	*Helianthus annuus* L.	Asteraceae	Mar.–May.	P, N
3	Cauliflower	*Brassica oleracea* L. var. *botrytis*	Brassicaceae	Dec.–Feb.	P, N
4	Chinese cabbage	*Brassica chinensis* L.D.	Brassicaceae	Dec.–Feb.	P, N
5	Edible leafy mustard	*Brassica juncea* Czern. & Coss.	Brassicaceae	Dec.–Feb.	P, N
6	Radish	*Raphanus sativus* L.	Brassicaceae	Dec.–Feb.	P, N
7	Rape	*Brassica napus* L.	Brassicaceae	Dec.–Feb.	P, N
8	Salad rocket	*Eruca vesicaria* ssp. *sativa* Mills.	Brassicaceae	Dec.–Feb	P, N
9	Turnip	*Brassica rapa* L.	Brassicaceae	Dec.–Feb.	P, N
10	Toria	*Brassica campestris* L. var. *toria*	Brassicaceae	Dec.–Feb.	P, N
11	Apple gourd	*Praecitrullus fistulosus* (Stocks) Pangalo	Cucurbitaceae	Mar.–Nov.	P, N
12	Bath sponge	*Luffa cylindrica* L.	Cucurbitaceae	Mar.–Nov.	P, N
13	Bitter gourd	*Momordica charantia* L.	Cucurbitaceae	Mar.–Nov.	P, N
14	Bottle gourd	*Lagenaria siceraria* (Molina) Standl.)	Cucurbitaceae	Mar.–Nov.	P, N
15	Cucumber	*Cucumis sativus* L.	Cucurbitaceae	Mar.–Nov.	P, N
16	Muskmelon	*Cucumis melo* L.	Cucurbitaceae	Mar.–Nov.	P, N
17	Pumpkin	*Cucurbita moschata* Duchesne ex Poir.	Cucurbitaceae	Mar.–Nov.	P, N
18	Ribbed gourd	*Luffa acutangula* (L.) Roxb.	Cucurbitaceae	Mar.–Nov.	P, N
19	Summer squash	*Cucurbita pepo* L.	Cucurbitaceae	Mar.–Nov.	P, N
20	Pigeon pea	*Cajanus cajan* (L.) Millsp.	Fabaceae	Sep.–Oct.	P, N
21	Chickpea	*Cicer arietinum* L.	Fabaceae	Dec.–Feb.	P, N
22	Berseem/clover	*Trifolium alexandrinum* L.	Fabaceae	Mar.–May	P, N
23	Lucerne	*Medicago sativa* L.	Fabaceae	Mar.–Oct.	P, N
24	Fenugreek	*Trigonella foenum-graecum* L.	Fabaceae	Feb.–Mar.	P, N
25	Guava	*Psidium guajava* L.	Myrtaceae	Apr.–May	P, N
26	Amla	*Phyllanthus emblica* Gatertn	Phyllanthaceae	Apr.–May	P
27	Pearl millet	*Pennisetum glaucum* (L.) R. Br.	Poaceae	Aug.–Sep.	P
28	Peach	*Prunus persica* (L.) Stokes	Rosaceae	March	P, N
29	Kinnow	*Citrus nobilis* × *Citrus deliciosa*	Rutaceae	Feb.–Mar.	P, N
30	Lemon	*Citrus limon* (L.) Burm. f.	Rutaceae	Feb.–Mar.	P, N
31	Coriander	*Coriandrum sativum* L.	Umbelliferae	Feb.–Mar.	P
32	Fennel	*Foeniculum vulgare* L.	Umbelliferae	Feb.–Mar.	P

N—Nectar source; P—Pollen source (here, the bee acts as the pollinator of the visited plant).

**Table 3:** Number of wooden planks occupied by migratory swarms of *A dorsata* under various treatments (1984–1993).

Hanging State of Wooden Planks	Treatment	
	Planks Traeted with Wax	Untreated Planks
Tightly Hung	29 (58)	0 (0)
Loosely Hung	0 (0)	0 (0)

Figures in the parentheses represent percent values.

**Table 4:** Occupancy level of various available nesting planks that were made available over the years to attract migratory swarms of *A. dorsata*.

Year	Nesting Plank Number and Occupation Status					
	1	2	3	4	5	6
1994	+	+	-	+	-	-
1995	+	-	+	+	-	-
1996	+	+	-	+	+	-
1997	+	-	-	+	+	-
1998	+	+	+	+	-	+
1999	+	+	-	+	-	-
2000	+	-	+	+	+	-
2001	+	+	-	+	-	+
2002	+	-	+	+	+	-
2003	+	+	-	+	+	-
2005	+	-	-	+	-	-
2006	+	+	-	+	+	-
2007	+	-	+	+	+	-
2008	+	+	+	+	-	+
2009	+	+	-	+	-	-
2010	+	-	-	+	+	-
2011	+	-	-	+	+	-
2012	+	+	-	+	-	+
Occupation Index	1	1	0.31	1	0.47	0.21
Re-occupation index	1	0.5	0.05	1	0.22	0

+ sign indicates occupation of the plank; - sign indicates that the plank was not occupied.

**Table 5:** Aggressive response of *A. dorsata* test colonies under two trial types after various durations of their settlements and pre-disturbance status.

No.	Days After Settlement the Handling Was Conducted	Disturbed Colonies *	Undisturbed Colonies *
1	10 (1st)	5,5,5,5,5	5,5,5,5,5
2	20 (2nd)	4,4,4,4,4	5,5,5,5,5
3	30 (3rd)	3,3,3,3,3	5,5,5,5,5
4	40 (4th)	2,2,2,2,2	5,5,5,5,5
5	50 (5th)	2,2,2,2,2	5,5,5,5,5
	Mean ± s.d.	3.2 ± 1.166	5.0 ± 0.0 *

* Data represent aggressiveness score; a score of 4 was assigned when the response was between 5 and 3; a score of 2 was assigned when the response was between 3 and 1 (see Table 1). Difference between disturbance trials: *p* < 0.0001, *t*-test value = 7.56; df = 48; Difference between days (Disturbed colonies): F_4, 24_ = ∞; Difference between days (Undisturbed colonies): Not applicable.

**Table 6:** Aggressive response of two types of *A. dorsata* test colonies subjected to four kinds of pre-handling treatments.

S. No.	Colony Status	Pre-Handling Treatment				
		Water	Smoke	Sham	Control	Average
1	Undisturbed	0,0,0,0,0	1,1,1,1,1	4,4,4,4,4	5,5,5,5,5	2.5
2	Periodically disturbed	0,0,0,0,0	0,0,0,0,0	3,3,3,3,3	4,4,4,4,4	1.75
Average		0	0.5	3.5	4.5	

Five colonies were used for each treatment. Data show the response of five colonies in each cell. Difference between disturbance status: *p* > 0.05, *t*-test value = 0.238, df = 38. Difference between taming trials—Disturbed colonies: F_3, 19_ = ∞; Undisturbed colonies: F_3, 19_ = ∞; a score of 4 was assigned when the response was between 5 and 3 (see Table 1).

**Table 7:** Yearly production of honey and beeswax by the giant honey bee (*A. dorsata*).

Colony. No.	Honey Production (kg)	Wax Production (kg)
1	6.8	1.5
2	7.2	1.6
3	5.8	1.2
4	6.4	1.7
5	6.3	1.6
6	5.9	1.5
7	7.1	1.8
8	6.2	1.4
9	7.5	1.6
10	6.6	1.3
Mean	6.58	1.52
Standard Deviation	0.53	0.18

## Data Availability

Data are available upon request from the author.
